# An Equivocal SCC Lesion—Antiepileptic-Induced CLOCC

**DOI:** 10.3390/brainsci12030384

**Published:** 2022-03-13

**Authors:** Maryla Kuczyńska, Monika Zbroja, Weronika Cyranka, Izabela Halczuk, Ewa Kopyto, Iwona Halczuk, Anna Drelich-Zbroja

**Affiliations:** 1 Department of Interventional Radiology and Neuroradiology, Medical University of Lublin, 20-059 Lublin, Poland; zbroanna@interia.pl; 2 Students’ Scientific Society at the Department of Interventional Radiology and Neuroradiology, Medical University of Lublin, 20-059 Lublin, Poland; m.zbroja8888@gmail.com (M.Z.); weronika.cyranka@gmail.com (W.C.); halczuk.izabela@gmail.com (I.H.); ewa.kopyto@gmail.com (E.K.); 3 Department of Neurology, Medical University of Lublin, 20-059 Lublin, Poland; iwonahal@gmail.com

**Keywords:** corpus callosum, splenium, magnetic resonance imaging, cytotoxic edema, epilepsy

## Abstract

We present a case of a woman who reported to the emergency unit due to recurrent episodes of severe headache and collapse. MRI examination revealed no relevant findings apart from small meningioma of the right parietal region. The patient was diagnosed with epilepsy and received outpatient treatment, which was changed due to poor toleration. A follow-up MRI was performed which revealed an isolated, focal lesion of the splenium of the corpus callosum. The patient underwent extensive laboratory testing and antiseizure medications were started again. Another MRI indicated substantial regression of the splenium of the corpus callosum (SCC) lesion. Both the complete clinical image and results of the diagnostic evaluation spoke in favor of cytotoxicity of the corpus callosum associated with anti-epileptic drug treatment. Pathologies involving the corpus callosum include congenital, demyelination, infection, neoplasm, trauma and vascular changes. Isolated, non-specific lesions of the splenium of corpus callosum usually indicate multiple sclerosis; however, other pathologies should be considered. Anti-epileptic drugs may evoke cytotoxic lesions of the corpus callosum (CLOCCs).

## 1. Introduction

The splenium of the corpus callosum (SCC) is the posterior end of the corpus callosum (CC) and until now, its exact function has not been fully elucidated. However, it connects the posterior cortices with fibers varying in size from thin late-myelinating axons in the anterior part, predominantly connecting parietal and temporal areas, to thick early-myelinating fibers in the posterior part, linking primary and secondary visual areas [1]. Numerous diseases can involve the SCC, resulting in diverse symptomatology, i.e., confusion, ataxia, dysarthria, and seizure [2]. Among them, there is reversible splenial lesion syndrome (RESLES)—a disorder characterized by the presence of a focal lesion usually localized within the central area of the splenium of the corpus callosum, which resolves on magnetic resonance imaging (MRI) after a period of time [3]. The etiology is diverse, from infections (streptococcus pneumoniae, meningococcal meningitis), metabolic conditions (hypoglycemia, hypernatremia), malnutrition, vitamin B12 deficiency, Kawasaki disease, malignancy, seizures and withdrawal of antiepileptic treatment [4]. Due to the rarity of the condition, large series have not been reported to unequivocally elucidate the nature of these lesions. Therefore, both the MR image and clinical manifestation can be challenging for clinicians.

Hereby, we present a case of a young female patient with epilepsy in whom an equivocal lesion of the SCC was found in magnetic resonance imaging in the course of anti-seizure therapy. Consecutive modifications of the antiepileptic treatment resulted in complete regression of the lesion on MRI. No alarming clinical symptoms have been reported by the patient since then.

## 2. Case Presentation

**Figure 1:**
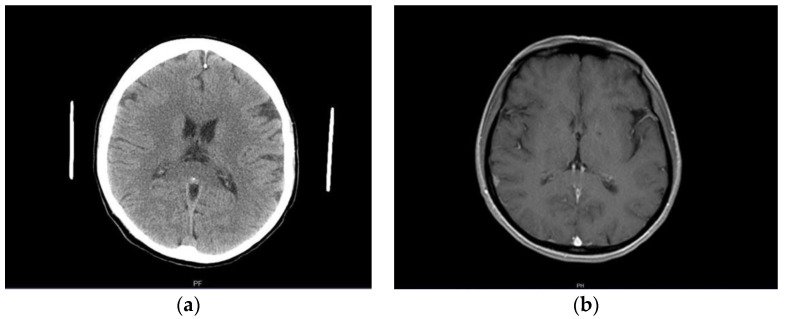
(**a**) Normal brain image—CT examination in native phase; (**b**) contrast-enhanced T1-weighted axial MR cross-section indicating a small right parietal meningioma (arrow).

A previously healthy female patient, age 41, reported to the emergency unit (June 2019) due to recurrent episodes (2–3× per week) of severe headache and collapse. She mentioned first symptoms occurring a month before admission (May 2019). No abnormalities were observed in neurological examination or brain computed tomography (CT) at the time (Figure 1a). Outpatient MRI examination was performed 2 months afterwards (August 2019), which revealed no relevant findings apart from a small (size < 1 cm) focal lesion, consistent with a meningioma of the right parietal region (Figure 1b). Next, the patient underwent electroencephalography (EEG) in October 2019, which indicated secondary generalized seizures originating bilaterally in the temporal lobes. Based on the clinical image, the patient was diagnosed with structural epilepsy and received outpatient treatment (400 mg carbamazepine twice daily). As the treatment was poorly tolerated by the patient, who felt weak, generally unwell, and reported worsening headaches as well as repeated episodes of collapse, a decision was made to substitute carbamazepine with lamotrigine (100 mg twice daily). No symptom alleviation was observed. In fact, the patient reported intensification of headache and general weakness. The drug was discontinued (beginning of June 2020). Due to repeated episodes of collapse, a cardiological consultation was made which excluded cardiogenic causes of the clinical image. The patient observed further exacerbation of clinical symptoms: headaches appeared on a daily basis; the patient collapsed with an average frequency of four times per month (with brief loss of consciousness at times); no convulsions or involuntary urination were present. Apart from hypoesthesia on the left side of the body, confirmed by neurological examination, no other abnormalities were found at the time.

**Figure 2:**
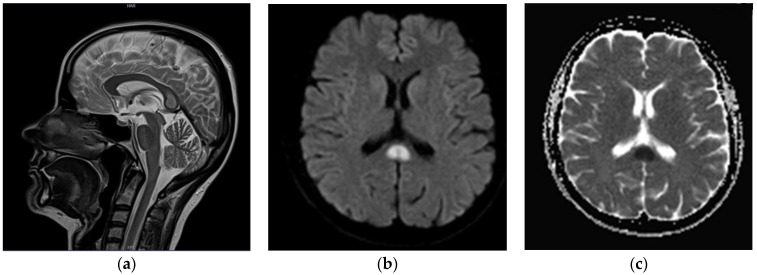
(**a**) T2-weighted (blade) images in sagittal plane; (**b**) DWI b = 1000 axial images; (**c**) corresponding Apparent Diffusion Coefficient (ADC) map. Well-demarcated, T2 hyperintense lesion (15 × 10 × 15 mm) within splenium of the corpus callosum (**a**,**b**) with apparent diffusion restriction on DWI/ADC images (**c**).

A follow-up head MRI was performed (end of June 2020), which revealed an isolated, focal lesion of the SCC (15 × 10 × 15 mm) that was hyperintense on T2w images, with diffusion restriction on diffusion-weighted images; no pathological contrast enhancement was seen (Figure 2). A differential diagnosis of the lesion comprised: active demyelinating plaque, Marchiavafa-Bignami syndrome (MBD) and ischemic lesion. The patient underwent extensive laboratory testing, including a coagulation panel, vitamin B12 concentration, electrolytes and inflammatory markers; no abnormalities were found (Table 1). She denied excessive alcohol consumption. The diagnosis of symptomatic epilepsy was maintained in the light of the above (2 July 2020); antiseizure medications, in particular valproic acid (target dose 300 mg twice daily), were started again. 

**Figure 3:**
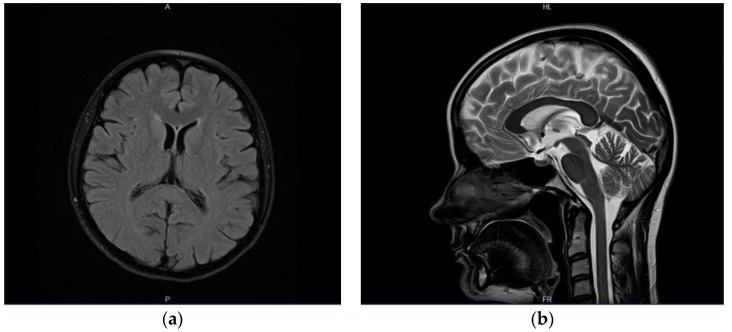
Another follow-up MR examination after 13 months. Substantial regression of the SCC lesion; discrete, indistinct hyperintensity on axial T2w-TIRM (dark fluid) images (**a**). No signal abnormalities were visible within splenium on other MR sequences—as visible on conventional T2-weighted (blade) sagittal image (**b**).

Furthermore, a follow-up MRI with spectroscopy was scheduled in 4–6 weeks to further evaluate the nature and metabolites of the SCC lesion. Consecutive neurological consultation revealed normal neurological status (9 July 2020); the patient did not report episodes of collapse since the previous evaluation. MRI (end of July 2020) indicated substantial regression of the SCC lesion (Figure 3); MR spectroscopy was not performed due to technical reasons (small residual foci). The patient was consulted by a neurosurgeon who did not see any indications for surgical intervention. Only three brief episodes of collapse without loss of consciousness or convulsions were reported by the patient during the consecutive month; minor, occasional concentration deficits were observed as well. A subsequent neurological examination revealed no abnormalities; therefore, treatment with valproic acid at a therapeutic dose of 2 × 300 mg was maintained. The last head MRI (February 2021, 7–8 months after the diagnosis of the lesion) showed complete resolution of the pathological foci within the SCC; there was no change in the appearance of the right parietal meningioma across all consecutive follow-up MR examinations. The neurological status of the patient was normal except for insignificant concentration disorders. Her mood improved markedly. Both the complete clinical image and results of the extensive diagnostic evaluation spoke in favor of a final diagnosis consistent with cytotoxic changes of the corpus callosum (CLOCC) associated with anti-epileptic drug treatment.

## 3. Discussion

In case of isolated, non-specific lesions of the SCC in diagnostic imaging, the following differential diagnosis should be considered:

− demyelinating lesions in the course of multiple sclerosis (MS);
− Marchiavafa-Bignami syndrome—pathomechanism: toxic effect of alcohol, electrolyte and osmotic disturbances, malnutrition and vitamin deficiencies;
− inflammatory involvement;
− neoplastic tumors (including lymphomas);
− Susac syndrome (autoimmune process initiating inflammatory changes and obstruction of cerebral capillaries) [5].

From the aforementioned, involvement of the corpus callosum and pericallosal area in the course of multiple sclerosis is the most common [1]. However, no abnormalities in neurological examination were observed that could indicate the diagnosis of MS; both the clinical image (recurrent headaches, collapses, brief loss of consciousness) and EEG results were consistent with epilepsy. For this reason, no further diagnostics for MS were carried out at the time and the patient was referred to the outpatient clinic. Symptomatology of the disease was inconsistent with clinical image of MS even at the time of MRI examination, which revealed an isolated lesion in the splenium of the corpus callosum (June 2020, >1 year from the onset of symptoms). Laboratory tests for Lyme disease, electrolyte deficiencies, and coagulation disorders did not reveal any abnormalities either. The cerebrospinal fluid examination for MS (IgG index and oligoclonal bands) was not performed for two reasons. Firstly, morphology of the SCC lesion could suggest a proliferative process, which is a contraindication of lumbar puncture; according to the MENACTRIMS (Middle East North Africa Committee for Treatment and Research in Multiple Sclerosis) recommendations for the diagnosis and treatment of MS, a follow-up neuroimaging examination and neurosurgical evaluation would be a better solution in such a case [6]. Furthermore, a lack of clinical signs typical of a demyelinating process dictates a wait-and-see attitude—waiting for the appearance of relapses, new clinical symptoms of the disease or subsequent demyelinating foci, even in the presence of an isolated non-specific demyelinating lesion on MRI. Only such policy warrants implementation of MS-specific treatment [6]. In addition, the patient did not provide consent for hospital admission due to the COVID-19 pandemic. Due to regression of both the clinical and imaging symptoms, there was no such absolute necessity. 

Marchiavafa-Bignami syndrome is a rare disorder of the central nervous system that is strongly associated with excessive alcohol consumption [7]. It involves acute demyelination and necrosis of the corpus callosum, most commonly observed in the CC body. Correlation between demyelination and CC lesions and other toxins (e.g., carbon monoxide) or psychoactive substances (e.g., cocaine, heroin) was observed as well [8]. The presented patient did not exhibit any electrolyte or osmotic abnormalities; malnutrition and vitamin deficiency were excluded; she denied alcohol or drug abuse, which was confirmed by her colleagues and family. Therefore, a diagnosis of MBD did not seem correct. 

Metabolic dysfunction, inflammatory process or traumatic brain injury may also result in CNS demyelination involving CC. Diffuse axonal injury is a common consequence of traumatic brain injury that frequently involves the parasagittal white matter, corpus callosum and brain stem [7]. However, such a background was excluded in the above case. Moreover, the patient did not have any history of blood glucose disturbances or diabetes; her blood glucose measurements were within normal limits, and to her knowledge, she did not experience states of hypoglycemia prior to admission. Throughout the outpatient diagnosis and hospitalization, the patient did not exhibit any signs of viral infection—she was not feverous at any stage of the disease and her laboratory parameters were normal (CRP, blood count).

Isolated SCC lesions may represent neoplastic infiltration as well. The most prevalent tumors found in the corpus callosum are glioblastomas, lymphomas and metastases [9]. Glioblastomas are the most common among astrocytomas, representing 50–60% of all cases. They frequently affect the population between 45 and 70 years of age [9]. Primitive cerebral lymphomas represent 1–7% of all brain tumors. They are usually non-Hodgkin B-cell tumors, and in recent years have been observed mostly in patients with a suppressed immune system or immunodeficiencies [10]. However, no disease progression was seen in our patient on diagnostic imaging. In fact, the MRI study revealed substantial regression of the SCC lesion after 5 weeks since the diagnosis, and complete regression in a follow-up study performed after 1 year. The patient received only anti-epileptic treatment during the whole period. 

Cytotoxic lesions of the corpus callosum (CLOCCs) are a result of an inflammatory cascade of changes manifested as cytotoxic edema secondary to an increased influx of water molecules in astrocytes and neurons caused by the release of the inflammatory cytokines interleukin-1 and interleukin-6 [11]. They have a characteristic MR image of intramyelinic edema that has a predilection for the corpus callosum. CLOCCs are secondarily associated with a variety of entities, including drug therapy, malignancies, infections, subarachnoid hemorrhages, metabolic disorders, trauma, and others [12]. Moreover, they were initially related to seizures; however, the cytotoxic edema can only partially influence the seizures because drug therapy can also cause CLOCCs [13]. Therapy with antiseizure drugs such as carbamazepine can influence fluid balance systems (arginine vasopressin) and influence proinflammatory and proconvulsive cytokines. It is stated that CLOCCs often develop after withdrawal of therapy with antiseizure drugs [12]. The lesion in the splenium of the corpus callosum regressed, which coincided with short-period cessation and further modification of the anti-epileptic treatment from lamotrigine to valproic acid. In the presented case, drug levels were not measured, due to the fact that patient’s seizure episodes were reduced and there were no adverse drug reactions. Both carbamazepine and lamotrigine, but also the withdrawal of anti-epileptic drugs may be the cause of reversible cytotoxic changes in the corpus callosum and this cause was diagnosed in our patient. Although CLOCCs are usually associated with reversible neurological signs, there are reports of permanent and irreversible damage of the corpus callosum [12,14].

## 4. Conclusions

− Pathologies involving the corpus callosum include congenital, demyelination, infection, neoplasm, trauma and vascular changes.
− Isolated, non-specific lesions of the SCC usually indicate multiple sclerosis; however, other pathologies such as CLOCC should be considered.
− Anti-epileptic drugs may be the cause of cytotoxic lesions of the corpus callosum.

## Figures and Tables

**Table 1:** Relevant laboratory indices of the patient.

	Value	Normal Range	Units
Creatinine	0.6	0.6–1.3	mg/dL
eGFR	>=90	>60	mL/min/1.73 m^2^
Folic acid	18.56	1.80–9.00	ng/mL
Borreliosis—IgM a/b	8.5 (negative)	<18.0	AU/mL
Borreliosis—IgG a/b	<5.0 (negative)	<5.0	AU/mL
Vitamin B12	272	211–911	pg/mL
D-dimers (G29)	164	<500	ng/mL
INR	1.0	0.8–1.2	-
Prothrombin index	95.6	70.0–130.0	%
Prothrombin time	11.4	12.0–16.0	s
Kaolin clotting time	29.4	26.0–40.0	s
Glucose (venous blood, serum)	95	70–99	mg/dL
Serum sodium	141	135–145	mmol/L
Serum potassium	4.0	3.5–5.0	mmol/L
C-reactive protein (CRP)—quantitative	2.950	<5.000	mg/L
Serum urea	23.60	15.00–40.00	mg/dL
Leukocytes (WBC)	6.47	3.50–9.00	×10^9^/L
Erythrocytes (RBC)	4.19	4.20–5.40	×10^12^/L
Hemoglobin (HGB)	12.9	11.5–16.0	g/dL
Hematocrit (HCT)	37.6	37.0–47.0	%
Platelets (PLT)	158	130–450	×10^9^/L

## Data Availability

The data that support the findings of this study are available on reasonable request from the corresponding author. The data are not publicly available due to privacy.
